# The plastome reveals new insights into the evolutionary and domestication history of peonies in East Asia

**DOI:** 10.1186/s12870-023-04246-3

**Published:** 2023-05-08

**Authors:** Qihang Chen, Le Chen, Jaime A. Teixeira da Silva, Xiaonan Yu

**Affiliations:** 1 College of Landscape Architecture, Beijing Forestry University, Beijing, 100083 China; 2 Beijing Key Laboratory of Ornamental Plants Germplasm Innovation and Molecular Breeding, Beijing, 100083 China; 3 National Engineering Research Center for Floriculture, Beijing, 100083 China; 4 Beijing Laboratory of Urban and Rural Ecological Environment, Beijing, 100083 China; 5 Independent researcher, Ikenobe 3011–2, Kagawa–ken, 761–0799 Japan

**Keywords:** Comparative genomics, Cultivar, *Paeonia*, Phylogenetics, Plastome

## Abstract

**Backgroud:**

*Paeonia* holds considerable value in medicinal, ornamental horticultural, and edible oil industries, but the incomplete state of phylogenetic research in this genus poses a challenge to the effective conservation and development of wild germplasm, and also impedes the practical utilization of existing cultivars. Due to its uniparental inheritance and lack of recombination, the plastome (i.e., plastid genome), which is a valuable molecular marker for phylogenetic analyses, is characterized by an appropriate rate of nucleotide evolution.

**Methods:**

In this study, 10 newly assembled data and available reported data were combined to perform a comparative genomics and phylogenetics analysis of 63 plastomes of 16 *Paeonia* species, primarily from East Asia, which is the origin and diversity center of *Paeonia*.

**Results:**

Ranging between 152,153 and 154,405 bp, most plastomes displayed a conserved structure and relatively low nucleotide diversity, except for six plastomes, which showed obvious IR construction or expansion. A total of 111 genes were annotated in the *Paeonia* plastomes. Four genes (*rpl22*, *rps3*, *rps19* and *ycf1*) showed different copy numbers among accessions while five genes (*rpl36*, *petN*, *psbI*, *rpl33* and *psbJ*) showed strong codon usage biases (ENC < 35). Additional selection analysis revealed that no genes were under positive selection during the domestication of tree peony cultivars whereas four core photosynthesis-related genes (*petA*, *psaA*, *psaB* and *rbcL*) were under positive selection in herbaceous peony cultivars. This discovery might contribute to the wide adaption of these cultivars. Two types of molecular markers (SSR and SNP) were generated from the 63 plastomes. Even though SSR was more diverse than SNP, it had a weaker ability to delimit *Paeonia* species than SNP. The reconstruction of a phylogenetic backbone of *Paeonia* in East Asia revealed significant genetic divergence within the *P. ostii* groups. Evidence also indicated that the majority of *P. suffruticosa* cultivars had a maternal origin, from *P. ostii*. The results of this research also suggest that *P. delavayi* var. *lutea*, which likely resulted from hybridization with *P. ludlowii*, should be classified as a lineage within the broader *P. delavayi* group.

**Conclusions:**

Overall, this study’s research findings suggest that the *Paeonia* plastome is highly informative for phylogenetic and comparative genomic analyses, and could be useful in future research related to taxonomy, evolution, and domestication.

**Supplementary Information:**

The online version contains supplementary material available at 10.1186/s12870-023-04246-3.

## Introduction

The genus *Paeonia*, which is famous for its application in medicinal, ornamental horticultural and edible oil industries, consists of about 33 species that are mainly distributed in temperate regions in Asia, Europe and North America [1]. The history of *Paeonia* domestication is at least 1500 years old, both in China and in Europe, and even though it was initially introduced from the wild for its medicinal uses, its ornamental value was rapidly discovered [1]. The most widely used active compounds in *Paeonia* species are paeonol and paeoniflorin, which are extracted from dried roots, and these have been extensively studied given their wide range of pharmacological activities [2]. To honor the economic and cultural value of this genus, traditional Chinese woody peony cultivars were crowned the “King of Flowers”, while Greek herbaceous cultivars were crowned as the “Queen of Herbs” [3]. Moreover, since the seeds of some *Paeonia* species contain high levels of unsaturated fatty acids, peony seed oil was approved in 2011 as a new food resource by the Chinese Ministry of Health (http://www.nhc.gov.cn/sps/). For these reasons, the planting area of oil peonies in China has increased steadily and is predicted to reach 5 million ha in the next 5–10 years [4].

The utilization of only a few *Paeonia* species is well developed but has been ignored in many others, most of them being endangered due to the pressures caused by urbanization and climate change [3]. In addition, the unresolved and complex nature of phylogenetic research in *Paeonia* prevents the expanded conservation and development of wild germplasm and hinders the utilization of existing cultivars [3]. As the origin and one of the centers of diversity of *Paeonia* [1], East Asia is vital to understanding the complete evolutionary history of this genus because it encompasses 18 species, 14 of which are endemic, and several thousand traditional herbaceous and woody cultivars [3]. Several studies have attempted to resolve the genetic relationships among species, and between species and cultivars [5–7], but several findings remain controversial. One of them is the *P. delavayi* complex, which formerly comprised several species (*P. lutea* Delavay ex Franch, *P. potaninii* Kom., and *P. trollioides* Stapf ex Stern), but is now considered a single species according to Hong’s taxonomy [8]. Over the past two decades, numerous studies were conducted on the molecular phylogenetics of *P. delavayi* [5–7]. Some research supports the monophyletic nature of *P. delavayi* [6, 9], but other studies have contradicted this claim, suggesting that *P. delavayi* may be paraphyletic [5, 10]. Each study may have limitations related to the molecular tool used (for instance, findings based on single or limited molecular fragments might not be representative of the entire genome) or sampling technique (e.g., from a single population, thus not representing the whole species), so the phylogenetics of *P. delavayi* remains inconclusive. To advance an understanding of *P. delavayi* and related issues in *Paeonia*, there is a pressing need for more comprehensive sampling and the utilization of a wider range of valid molecular tools in phylogenetic research.

The plastome (i.e., plastid genome) is a widely used and effective molecular marker for plant phylogenetic and evolutionary studies due to several advantageous features, including its abundance, the presence of single-copy genes, the absence of recombination, and a suitable rate of nucleotide evolution [11]. The *rbcL* gene, which was the first plastid gene to be widely used in plant phylogeny [12], has also been employed as a barcode for species identification [13]. However, due to a high level of conservation among closely related genera, additional plastome genes, such as *matK* and *rpoC*, as well as non-coding fragments such as introns or intergenic regions, have been used to improve the accuracy of phylogenetic analyses [13]. Unfortunately, the use of single or multiple fragments still cannot reveal variation among closely related species and this may introduce conflicting findings in phylogenetics [11]. The optimization of sequencing and assembling technologies has enabled phylogenetic analyses based on whole plastomes, demonstrating their powerful ability to resolve complicated phylogenetic relationships at the species or population level [14]. Previous phylogenetic research in *Paeonia* employed the whole plastome, but the sample size of these studies was limited. For instance, the most comprehensive study to date [10] included only 15 samples representing seven species. Likewise, other studies utilizing whole plastomes have displayed similar limitations due to small sample sizes, leading to conflicting results. Gao et al. [15] found that *P. brownii* was more closely related to sect. *Paeonia* than to sect. *Moutan*, while Dong et al. [16] reported opposite results. Thus, a more comprehensive plastome-based phylogenetic study is needed to provide new insight into the evolutionary history of *Paeonia*. Furthermore, some research indicated that domestication can lead to variation in plastomes between wild and cultivated materials, including the positive selection of genes and single nucleotide variants [17, 18]. However, comparative studies related to domestication are still lacking in *Paeonia*.

By comprehensively exploring available reported resources and targeted sampling to supplement new plastomes, we generated a complete dataset of peony plastomes in East Asia, including both natural and cultivated accessions. This dataset was subjected to phylogenetic and comparative genomic analyses to achieve three objectives: (1) to comprehensively identify variation in the structure and content of the *Paeonia* plastome as a way to evaluate the level of genetic variation in East Asia; (2) to conduct a comparative genomic analysis between cultivars and natural germplasm as a way to explore the domestication history of peony cultivars in East Asia; (3) to perform a phylogenetic analysis to resolve the evolutionary history of *Paeonia* species in East Asia.

## Materials and methods

### Plant materials, DNA sequencing and plastome assembly

Four wild accessions were collected from their natural habitats, including three accessions of *P. mairei* from Qinling Mountains, Daba Mountains and Hengdaun Mountains, which represent its three main regions of distribution [1], and one *P. sterniana* accession from southeastern Tibet where its distribution is limited [1]. All samples were identified and collected by the first author in 2020 and further checked by Yong Yang (Institute of Botany, the Chinese Academy of Sciences). Voucher specimens were preserved in Beijing Forestry University Museum (BJFC001128-BJFC001130 for *P. mairei* accessions from Qinling, Daba and Hengduan populations, BJFC001131 for the *P. sterniana* accession). Young leaves were dried over silica gel and total DNA was extracted from each accession using a modified CTAB method [19]. Genomic DNA was fragmented into 350–500 bp to construct libraries with the TruSeq DNA Sample LT Prep kit (Illumina, San Diego, CA, US) and sequenced on the Illumina NovaSeq platform.

**Fig. 1:**
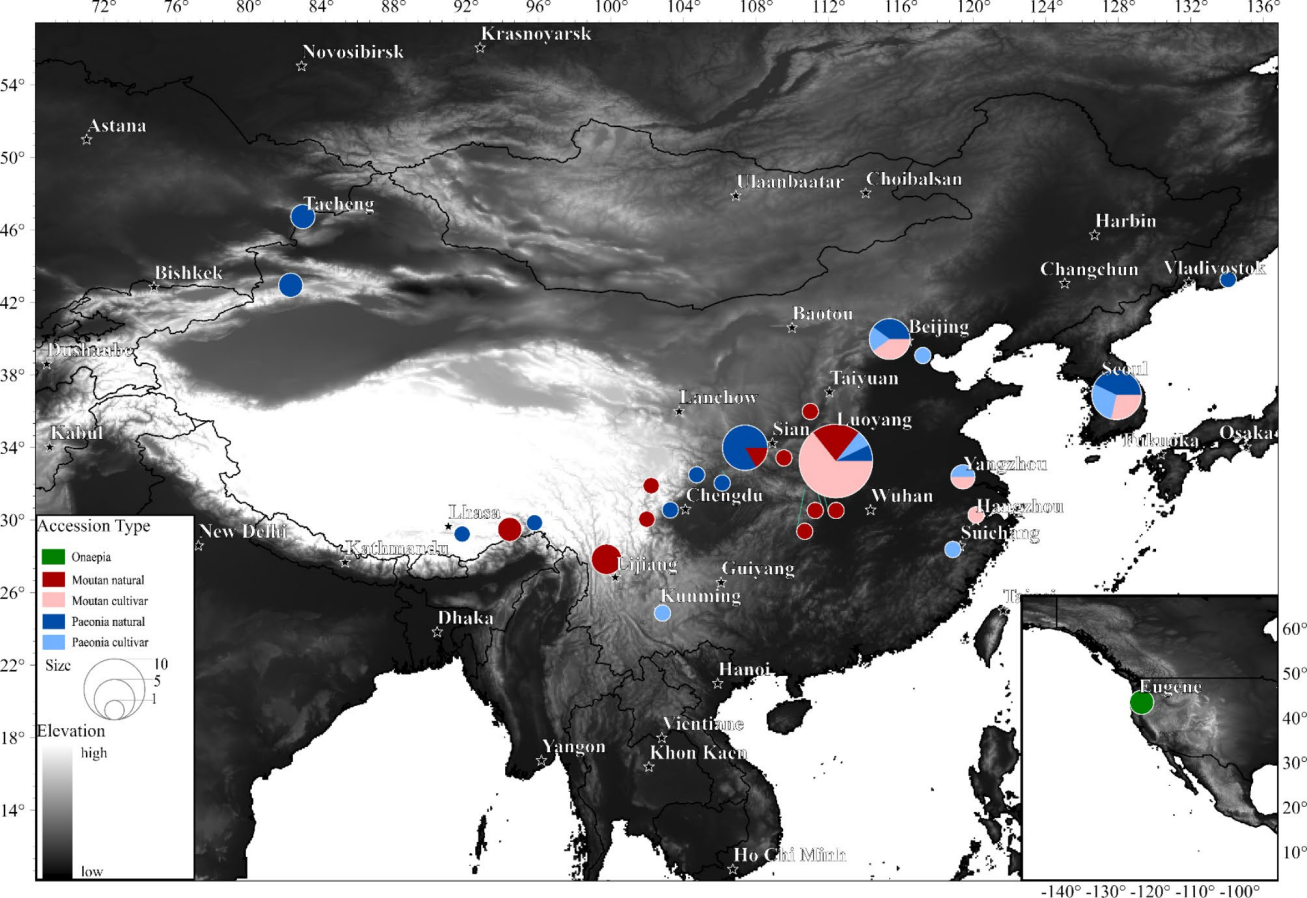
Geographic distribution and sample size of 63 *Paeonia* accessions analyzed in this research. The focus was on *Paeonia* germplasm in East Asia, but also included two accessions from North America to explore the genetic relationship between East Asian and North American peonies. The map was generated by ArcGIS Pro (Esri, Redlands), and elevation was illustrated according Harmonized World Soil Database (https://www.fao.org/soils-portal/data-hub/soil-maps-and-databases/harmonized-world-soil-database-v12)

Available datasets were searched in NCBI’s GenBank (www.ncbi.nlm.nih.gov/genbank/) and SRA (www.ncbi.nlm.nih.gov/sra/) databases in April 2022, and a total of 67 reported plastome assemblies and 17 whole genome sequencing (WGS) datasets were collected. Data were filtered out if they were duplicated or missing exact sample information. If both assembly and WGS data were available (seven accessions) for a sample, then only WGS data was retained to ensure consistency in assembly methods. The final dataset covered 46 plastomes and 17 WGS datasets, including four newly sequenced accessions. The data preparation pipeline is illustrated in Supplementary Fig. 1, and the full list of analyzed accessions is presented in Fig 1 and Table 1. The GetOrganelle pipeline [20], which was used to assemble all WGS data, has been shown to outperform other common assemblers in terms of consistency, accuracy, and success rate [20, 21]. In GetOrganelle, the automated pipeline of the *de novo* assembly was performed from reads using default settings. Finally, a total of 63 *Paeonia* plastomes, including of 23 cultivars and 40 natural accessions that cover 16 wild *Paeonia* species in East Asia, were available for downstream analysis (Fig. 1; Table 1). However, *P. cathayana*, which is now considered to be extinct in the wild [1], was not included in the analysis. Two *P. brownii* accessions were also included, allowing the genetic relationship between peonies from East Asia and North America to be explored. Hong’s taxonomy [1], which divides all *Paeonia* species into three sections (sect. Onaepia, Moutan, and Paeonia), was used in this research. Specifically, sect. Onaepia includes two species that are endemic to North America, sect. Moutan includes eight species that are endemic to East Asia, and sect. Paeonia includes 23 species that are widely distributed throughout Eurasia. To compare plastome variation and genetic diversity among the three sections, as well as between cultivars and natural accessions, the 63 accessions were manually clustered into five groups (Table 1): ONAE (N = 2), MOUT_WILD (N = 17), MOUT_CULT (N = 15), PAEO_WILD (N = 21), and PAEO_CULT (N = 8).

**Table 1:** Accessions analyses in the present research

Group	ID	Species	Section	Infra species	Cultivar	Life type	Sample region	Assembly accession	SRA accession
ONAE	brownii_1	*P. brownii*	*onaepia*		no	herbaceous	California, USA	JQ952560	
	brownii_2	*P. brownii*	*onaepia*		no	herbaceous	California, USA	MH191385	
MOUT_WILD	decomposita_1	*P. decomposita*	*moutan*		no	woody	Sichuan, China	MG571273	
	delavayi_1	*P. delavayi*	*moutan*		no	woody	Xizang, China	KY817591	
	delavayi_2	*P. delavayi*	*moutan*	var. *lutea*	no	woody	Yunnan, China	MK701989*	
	delavayi_3	*P. delavayi*	*moutan*	var. *potaninii*	no	woody	Yunnan, China	MK701991	
	delavayi_4	*P. delavayi*	*moutan*		no	woody	Yunnan, China	MN463100	
	delavayi_5	*P. delavayi*	*moutan*	var. *lutea*	no	woody	Yunnan, China	MT210546	
	jishanensis_1	*P. jishanensis*	*moutan*		no	woody	Henan, China	MT210545	
	jishanensis_2	*P. jishanensis*	*moutan*		no	woody	Shaanxi, China	MG991935	
	jishanensis_3	*P. jishanensis*	*moutan*		no	woody	Shanxi, China	MK701988	
	ludlowii_1	*P. ludlowii*	*moutan*		no	woody	Xizang, China	KY817592	
	ostii_1	*P. ostii*	*moutan*		no	woody	Henan, China	MK701990	
	ostii_2	*P. ostii*	*moutan*		no	woody	Henan, China	OM179763	SRR19122852
	ostii_3	*P. ostii*	*moutan*		no	woody	Henan, China	OM291541	SRR19122851
	qiui_1	*P. qiui*	*moutan*		no	woody	Hubei, China	MK701992	
	qiui_2	*P. qiui*	*moutan*		no	woody	Hubei, China	MT210544	
	rockii_1	*P. rockii*	*moutan*	subsp. *rockii*	no	woody	Shaanxi, China	MF488719*	
	rockii_2	*P. rockii*	*moutan*	subsp. *avata*	no	woody	Shaanxi, China	MW192444	
MOUT_CULT	ostii_4	*P. ostii*	*moutan*	cv. FengDan	yes	woody	Henan, China	MG572457	
	ostii_5	*P. ostii*	*moutan*	cv. FengDan	yes	woody	Henan, China	MG585274	
	ostii_6	*P. ostii*	*moutan*	cv. FengDan	yes	woody	Henan, China	OP324591**	SRR6476733
	ostii_7	*P. ostii*	*moutan*	cv. FengDan	yes	woody	Jiangsu, China	OP324592**	SRR7614768
	rockii_3	*P. rockii*	*moutan*	cv. FenEJiao	yes	woody	Henan, China	MK701993	
	suffruticosa_1	*P. × suffruticosa*	*moutan*		yes	woody	Beijing, China	JQ952559	
	suffruticosa_2	*P. × suffruticosa*	*moutan*		yes	woody	Beijing, China	MH191384	
	suffruticosa_3	*P. × suffruticosa*	*moutan*	cv. DouLv	yes	woody	Henan, China	MK701994	
	suffruticosa_4	*P. × suffruticosa*	*moutan*	cv. LuoYangHong	yes	woody	Henan, China	MK701995	
	suffruticosa_5	*P. × suffruticosa*	*moutan*	cv. ShouAnHong	yes	woody	Henan, China	MK701996	
	suffruticosa_6	*P. × lemoinei*	*moutan*	cv. HighNoon	yes	woody	Henan, China	MK701997	
	suffruticosa_7	*P.* × *suffruticosa*	*moutan*		yes	woody	Incheon, South Korea	MH793271	
	suffruticosa_8	*P.* × *lemoinei*	*moutan*	cv. HwangMoran	yes	woody	Incheon, South Korea	MK860970	
	suffruticosa_9	*P. × suffruticosa*	*moutan*	cv. LianHe	yes	woody	Henan, China	OM179764	SRR19122853
	suffruticosa_10	*P. × suffruticosa*	*moutan*	cv. YuLuoChun	yes	woody	Zhejiang, China	OK662586*	
PAEO_WILD	anomala_1	*P. anomala*	*paeonia*		no	herbaceous	Xinjiang, China	MT210549	
	emodi_1	*P. emodi*	*paeonia*		no	herbaceous	Xizang, China	MT210548	
	intermedia_1	*P. intermedia*	*paeonia*		no	herbaceous	Xinjiang, China	MT210547	
	intermedia_2	*P. intermedia*	*paeonia*		no	herbaceous	Xinjiang, China	OP324584**	ERR3525038
	intermedia_3	*P. intermedia*	*paeonia*		no	herbaceous	Xinjiang, China	OP324585**	ERR3525039
	lactiflora_1	*P. lactiflora*	*paeonia*		no	herbaceous	Primorskiy Kray, Russian	MG897127	
	lactiflora_2	*P. lactiflora*	*paeonia*		no	herbaceous	Shaanxi, China	MN061945*	
	mairei_1	*P. mairei*	*paeonia*		no	herbaceous	Shaanxi, China	OP324589**	
	mairei_2	*P. mairei*	*paeonia*		no	herbaceous	Shaanxi, China	OP324590**	
	mairei_3	*P. mairei*	*paeonia*		no	herbaceous	Sichuan, China	MN508366*	
	mairei_4	*P. mairei*	*paeonia*		no	herbaceous	Sichuan, China	OP324588**	
	obovata_1	*P. obovata*	*paeonia*		no	herbaceous	Beijing, China	JQ952561	
	obovata_2	*P. obovata*	*paeonia*		no	herbaceous	Beijing, China	MH191383	
	obovata_3	*P. obovata*	*paeonia*		no	herbaceous	Chungcheongbuk-do, South Korea	KJ206533	
	obovata_4	*P. obovata*	*paeonia*		no	herbaceous	Chungcheongbuk-do, South Korea	MT821944*	
	obovata_5	*P. obovata*	*paeonia*		no	herbaceous	Chungcheongbuk-do, South Korea	MT821946	SRR13840229
	obovata_6	*P. obovata*	*paeonia*	subsp.*willmottiae*	no	herbaceous	Henan, China	MN149613	
	obovata_7	*P. obovata*	*paeonia*	subsp.*willmottiae*	no	herbaceous	Shaanxi, China	MN840851	
	sterniana_1	*P. sterniana*	*paeonia*		no	herbaceous	Xizang, China	OP324593**	
	veitchii_1	*P. veitchii*	*paeonia*		no	herbaceous	Shaanxi, China	KT894821	
	veitchii_2	*P. veitchii*	*paeonia*		no	herbaceous	Shaanxi, China	MW762596	SRR17202104
PAEO_CULT	lactiflora_3	*P. lactiflora*	*paeonia*	var.*trichocarpa*	no	herbaceous	Chungcheongbuk-do, South Korea	MT821945	SRR13840230
	lactiflora_4	*P. lactiflora*	*paeonia*		yes	herbaceous	Beijing, China	OP324586**	SRR15412863
	lactiflora_5	*P. lactiflora*	*paeonia*	cv. LvHe	yes	herbaceous	Henan, China	MN149612	
	lactiflora_6	*P. lactiflora*	*paeonia*		yes	herbaceous	Incheon, South Korea	MK860971	
	lactiflora_7	*P. lactiflora*	*paeonia*		yes	herbaceous	Jiangsu, China	OP324587**	SRR7614723
	lactiflora_8	*P. lactiflora*	*paeonia*		yes	herbaceous	Tianjin, China	MW762595	SRR17202105
	lactiflora_9	*P. lactiflora*	*paeonia*		yes	herbaceous	Yunnan, China	KF753636	
	lactiflora_10	*P. lactiflora*	*paeonia*		yes	herbaceous	Zhejiang, China	MN868412	

*, annotation reference

**, newly reported assembly

### Plastome annotation, characterization, and comparison

A total of six reported and annotated *Paeonia* plastomes (Table 1) were downloaded from NCBI and manually checked for the use of annotated references in this work. PGA.pl [22] was then applied for the annotation of elements (including inverted repeat boundaries, genes, CDS, tRNA and rRNA) on all plastomes. Repeat sequences were annotated using three methods. Tandem repeats in each plastome were identified using the Tandem Repeats Finder (TRF) [23] with default parameters. Microsatellites, also known as simple sequence repeats (SSR), were predicted using MISA [24] with repeat thresholds of ten for mononucleotides, five for di- and trinucleotide SRRs, and four for tetra-, penta- and hexanucleotides. Interspersed repetitive sequences (forward, reverse, complement, or palindromic) were identified using REPuter [25], with a maximum hamming distance set to three and the minimum repeat size set to 30 bp. SeqKit [26] was used to summarize the statistics of characteristics pertaining to all plastomes, including sequence length and GC content. All plastome features were compared among the five groups to identify variations in patterns. In this case, analysis of variance (ANOVA) was used to compare mean values, and coefficients of variation (CVs) were calculated in R version 4.1.3.

The codon usage bias of each coding sequence (CDS) was assessed using codonW [27]. Relative synonymous codon usage (RSCU) was used to determine the codon preference pattern. The effective number of codons (ENC) was used to evaluate the usage bias of a specific sequence. ENC ranges from 20 (indicating absolute bias, where each amino acid has only one valid codon) to 61 (indicating no bias, where all codons are used evenly) [28]. HyPhy software [29] was used to identify instances of diversifying and episodic selections in *Paeonia*. The fixed-effects likelihood (FEL) [30] and Fast Unconstrained Bayesian AppRoximation (FUBAR) [31] models were applied to infer the rates of non-synonymous (dN) and synonymous (dS) substitutions to identify instances of diversifying selection among all accessions. The MEME model [32] was used to identify episodic selection in two cultivar groups (MOUT_CULT and PAEO_CULT) using a mixed-effects maximum likelihood approach.

### Detection of genetic variation and diversity analysis

Plastome arrangement and structure variations were identified by multiple sequence alignment using Mauve [33] with default parameters. Since no plastome rearrangement was detected by Mauve, the 63 plastomes were further aligned in MAFFT [34] using the local pair mode. Sites with less than six accessions were trimmed by trimAl [35] to generate a high-quality sequence matrix. The nucleotide diversity index (Pi) along plastomes was calculated by a sliding window with a 1000-bp width and a 500-bp step using pegas [36] based on the sequence matrix. To understand the distribution pattern and diversity of nucleotides along the plastome, a consensus plastome was generated by the consensus function in the seqinr package [37] and annotated by PGA.pl [22].

Two types of molecular markers (SSR and single nucleotide polymorphorphisms (SNPs)) were also used to analyze genetic diversity and delimit species. To identify shared SSRs from plastome sequences, a 30-bp sequence upstream of each SSR, as detected by MISA.pl [24], was extracted and checked through a blast algorithm. SNPs were detected in the aligned plastome matrix using adegenet [38]. To control bias caused by sample size during the analysis of genetic diversity, eight accessions were randomly resampled from each group (ONAE was excluded as it only has two accessions). The genetic diversity based on each marker method was measured using Poppr [39]. A series of indexes was calculated, including *eMLG* (the expected number of multilocus genotypes in the lowest common sample size) to measure genotype abundance, three indexes to measure genotype diversity (*H*, Shannon-Weiner index; *G*, Stoddard and Taylor’s index; *λ*, Simpson’s index), and an index to measure genotype evenness (*E5*). Discriminant analysis of principal components (DAPC) [40] was used to identify genetic structures and potential key markers for distinguishing the five groups for each marker method in adegenet [38]. Dendrograms with bootstrap support were generated in Poppr for both SSR and SNP markers to assess their effectiveness in delimiting species. Nei’s distance method was applied for SSR and Hamming distance was used for SNP.

### Phylogeny based on whole plastomes

Given that *Paeonia* is distant from other lineages, having diverged from the nearest lineage in Saxifragales about 100 Ma ago [41], it was difficult to select a suitable outgroup for the *Paeonia* phylogeny. As an alternative, the direction of evolution was inferred using a Bayesian evolutionary analysis with BEAST [42]. BEAST can generate time-trees based on prior distributions of the tree (e.g. coalescent and birth–death families) [43]. The full plastome was divided into two partitions, coding and non-coding, to account for the different evolutionary pressures each experiences [17], and each partition was modeled separately using different parameters. To generate the coding partition, the protein-coding CDS for all accessions, which had been annotated by PGA.pl, were extracted by bedtools [44]. The resulting CDS sequences were then aligned and trimmed based on codons using prank [45] and trimAl [35]. To generate the non-coding partition, all coding sequences (including CDS, tRNA and rRNA) and repeat sequences (including tandem and interspersed) were masked from plastomes by bedtools, and the resulting masked plastomes were aligned by MAFFT [34] and trimmed by trimAl [35]. For each data partition (coding and non-coding), the best site model was selected using ModelFinder [46], which implements the Bayesian information criterion (BIC) to identify the best-fit substitution model. The best clock model and tree prior were identified by path sampling [47], which is a Bayesian method for model selection and averaging that estimates the marginal likelihood of competing models. Zhou et al. [7] proposed an age estimate for the crown group of *Paeonia* at 28 Ma based on fossil calibration points. Therefore, in the current study, the age of the MRCA was constrained to a normal distribution with a mean of 28 Ma, and the lower and upper limits were set at 25 Ma and 32 Ma, respectively. A Markov Chain Monte Carlo (MCMC) chain of 100 million generations was run, and samples were assessed every 2000th generation. The results were verified using Tracer [48] to ensure that the effective sample size (ESS) of each parameter was greater than 200. After discarding the first 20% of trees as burn-in using TreeAnnotator (in BEAST), a maximum clade credibility (MCC) tree was generated. The final phylogenetic tree with divergence time bars was plotted with ggtree [49]. A dataset of benthic δ ^18^O content [50], which is related to global paleoclimate, was downloaded and used to compare it with the rate of divergence branches in *Paeonia*. To examine the genetic structure of clusters generated by Bayesian phylogeny, a principle component analysis (PCA) based on SNPs generated in Sect. 1.3 was performed in adegenet.

## Results

### Features of the ***Paeonia*** plastome

#### Structure and size

In this research, a total of 17 complete plastomes were assembled and 10 newly reported plastomes were submitted to the NCBI database. The *Paeonia* plastome exhibits a quadripartite structure, consisting of two identical copies of the inverted repeat (IR) region separated by a large single copy (LSC) region and a small single copy (SSC) region. The plastome sequence length of the 63 accessions ranged from 152,153 to 154,405 bp, with an average length of 152,741 bp (Table 2). The length of the four regions among the five groups was analyzed by ANOVA. The results indicate that there was significant variation in SSC length among the groups (*p* < 0.01). The ONAE group had the shortest SSC (16,679 bp) while the *Paeonia* groups (16,969 bp in PAEO_CULT, 17,019 bp in PAEO_WILD) exhibited a shorter SSC region than *Moutan* groups (17,051 bp in MOUT_CULT, 17,045 bp in MOUT_WILD). The CV for the length of the IR region was higher (0.99%) than that of the LSC (0.33%) and SSC (0.41%) regions, and *Moutan* accessions showed a higher sequence length CV than *Paeonia* and *Onaepia* accessions (Table 2). The plastome GC content for all accessions ranged from 38.32 to 38.55%, with an average of 38.42% (Table 2). The IR region had a higher GC content (43.09%) than the LSC (36.71%) and SSC (32.70%) regions. There was significant variation in GC content among the groups (*p* < 0.01), regardless of whether this referred to the whole plastome or the four regions. *Onaepia* accessions exhibited the highest GC content (38.55%) while *Moutan* accessions showed the lowest value (38.36%).

**Table 2:** Sequence length and GC content of different *Paeonia* groups

	Group	Mean					CV			
		Plastome	LSC	SSC	IR		Plastome	LSC	SSC	IR
Sequence length	Whole	152,741 ± 301	84,402 ± 277	17,016 ± 69	25,662 ± 247		0.20%	0.33%	0.41%	0.99%
	ONAE	152,227 ± 1	84,261 ± 0	16,679 ± 0	25,644 ± 1		0	0	0	0
	MOUT_WILD	152,849 ± 441	84,330 ± 392	17,046 ± 22	25,736 ± 373		0.29%	0.47%	0.13%	1.46%
	MOUT_CULT	152,688 ± 290	84,530 ± 367	17,050 ± 12	25,554 ± 283		0.19%	0.44%	0.07%	1.14%
	PAEO_WILD	152,709 ± 60	84,383 ± 46	17,014 ± 20	25,656 ± 11		0.04%	0.06%	0.12%	0.04%
	PAEO_CULT	152,822 ± 236	84,400 ± 31	16,978 ± 23	25,722 ± 132		0.15%	0.04%	0.13%	0.51%
GC content	Whole	38.4 ± 0.05%	36.7 ± 0.05%	32.7 ± 0.09%	43.09 ± 0.07%		0.13%	0.13%	0.28%	0.17%
	ONAE	38.55 ± 0%	36.83 ± 0%	33.02 ± 0%	43.16 ± 0%		0	0	0	0
	MOUT_WILD	38.37 ± 0.04%	36.68 ± 0.04%	32.64 ± 0.07%	43.05 ± 0.11%		0.10%	0.12%	0.22%	0.26%
	MOUT_CULT	38.36 ± 0.04%	36.66 ± 0.03%	32.65 ± 0.06%	43.09 ± 0.03%		0.09%	0.10%	0.18%	0.07%
	PAEO_WILD	38.43 ± 0.01%	36.72 ± 0.01%	32.75 ± 0.04%	43.13 ± 0.02%		0.02%	0.03%	0.11%	0.05%
	PAEO_CULT	38.43 ± 0.01%	36.74 ± 0.01%	32.72 ± 0.01%	43.1 ± 0.05%		0.02%	0.03%	0.04%	0.11%

CV, coefficient of variation; IR, inverted repeat sequence; LSC, large single-copy sequence; SSC, small single-copy sequence

#### IR expansion and contraction

**Fig. 2:**
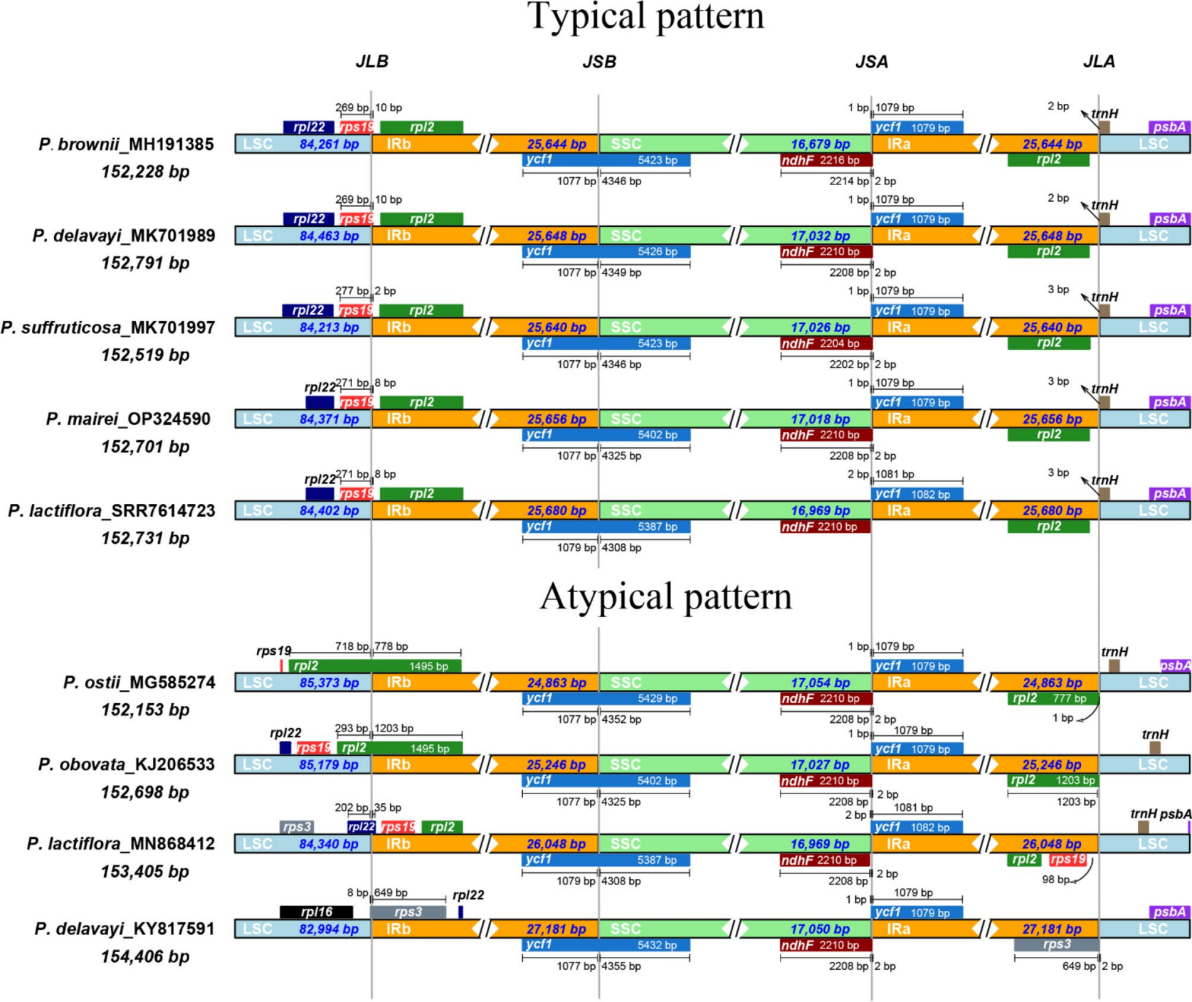
Distribution and structure of the inverted repeat region (IR) in *Paeonia* plastomes. Most plastomes have a conserved IR structure (typical pattern). Five accessions were selected to represent the five groups. Atypical patterns, i.e., IR expansion and contraction, were detected in several accessions

As indicated by the results above, the IR region exhibited higher variation in sequence length than the LSS and SSC regions. This variation may be attributed to the process of IR expansion and contraction, which often occurs due to changes in IR borders. The results show that the borders of two IRs (IRa and IRb) in *Paeonia* plastomes are conserved in most of the analyzed accessions (57/63). The typical junction pattern for most accessions was plotted in Fig. 2 (the complete version of all accessions can be observed in Supplementary Fig. 2), in which all of the LSC/IRb junctions fell into *rps19*, although the specific loci varied slightly. The IRb/SSC, SSC/IRa and IRa/LSC junctions fell into *ycf1*, *ndhF* and *trnH*, respectively, with slightly modified loci. While most accessions were conserved, significant expansion and contraction were observed in six accessions. Four accessions (*P. ostii* MG585274 and MG572457, *P. rockii* MF488719, and *P. obovata* KJ206533) showed IR contraction. In those cases, their LSC/IRb junctions fell into *rpl12* rather than *rps19*. In two accessions (*P. lactiflora* MN868412 and *P. delavayi* KY817591) that showed IR expansion, their IRs covered the entire *rpl19* gene.

#### Repeat sequences

**Fig. 3:**
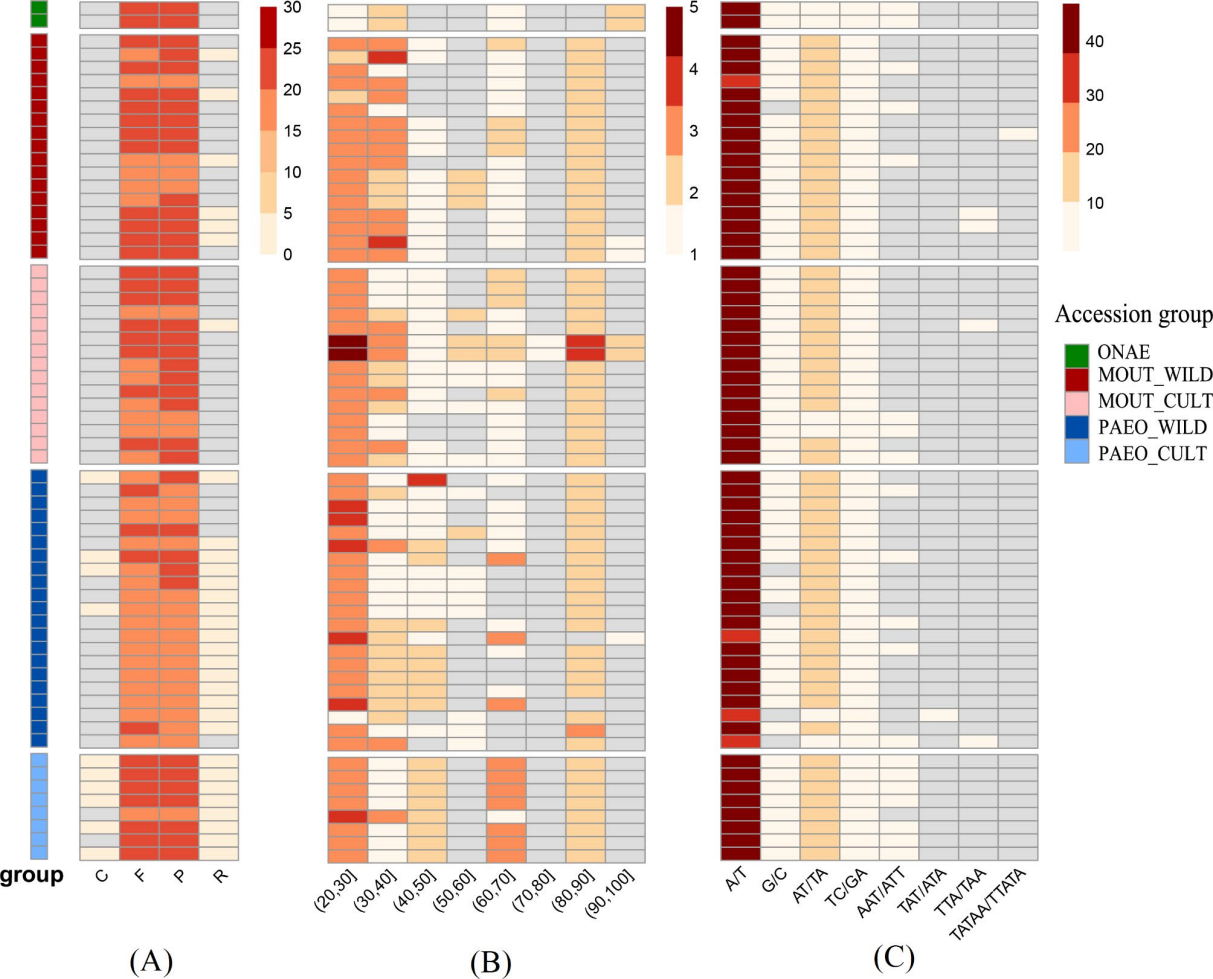
Abundance of repeat sequences annotated in *Paeonia* plastomes. (**A**) Interspersed repeat sequences (C, complement; F, forward; P, palindromic; R, reverse); (**B**) Long tandem repeat sequences; length range: 20 to 100; two regions of abundance (20–30, 80–90); (**C**) Short tandem repeat sequences, seven motifs were detected

REPuter detected forward (F) and palindromic (P) repeats in all accessions whereas inverted (R) and complementary (C) repeats were mainly detected in sect. *Paeonia*. Four types of interspersed repeats were most abundant in PAEO_CULT (Fig. 3A). Long tandem repeat sequences of varying length (20–100 bp) were identified by TRF, and there were two regions of abundance (20–30 and 80–90) (Fig. 3B). SSR detection results revealed the existence of eight motifs (including two monomeric, two dimeric, three trimeric and one pentameric repeats) in *Paeonia*, with monomeric repeats being the most abundant (Fig. 3C). Each accession contained 46–64 SSRs, 32 of which were shared among at least half of all accessions.

### Gene annotation and detection of features

#### Gene annotation

**Fig. 4:**
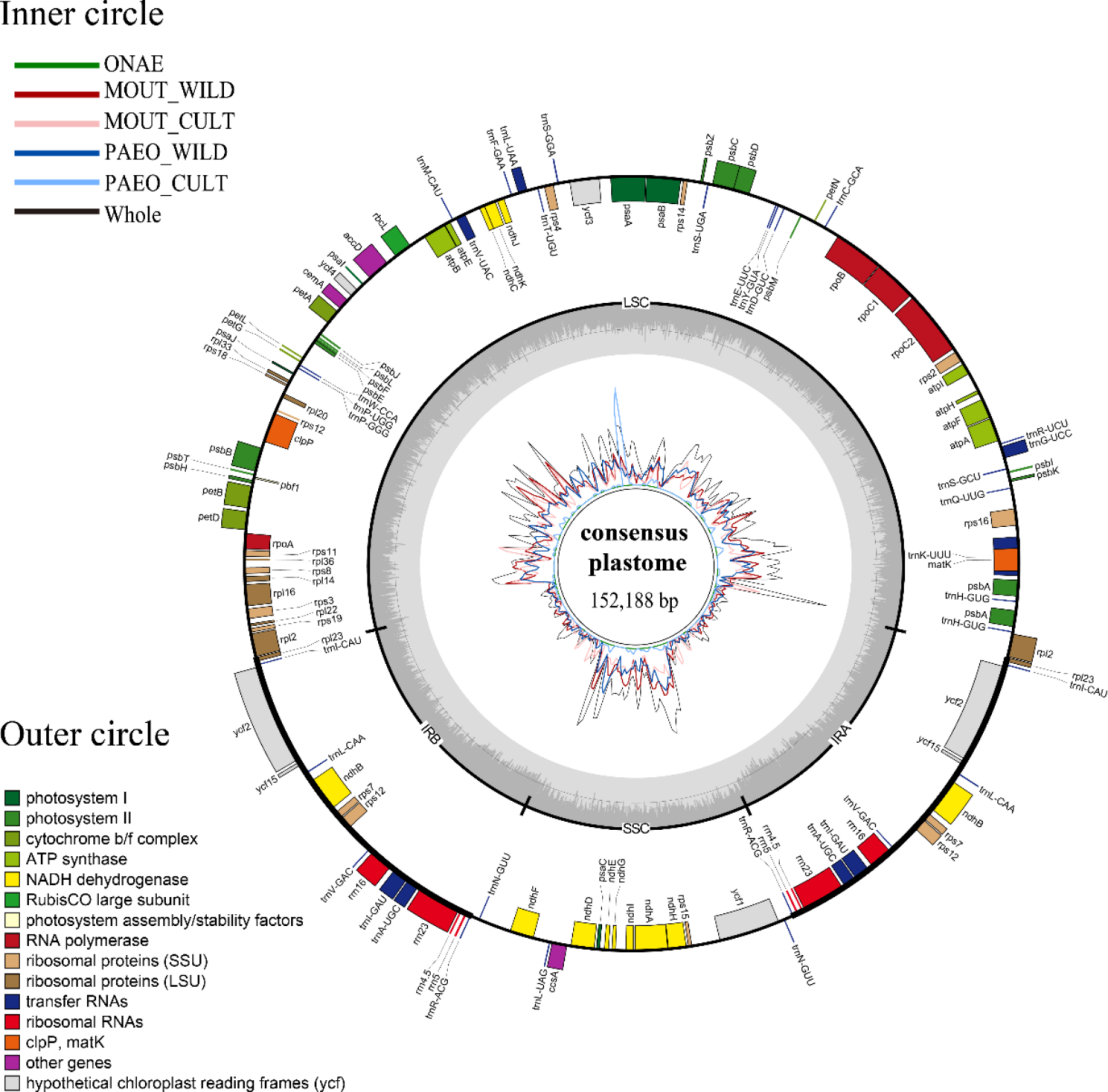
Distribution of annotated genes on the consensus plastome and nucleotide diversity along the plastome. A consensus plastome was generated by aligning and trimming 63 plastomes. The outer circle displays a distribution pattern of annotated genes on plastome genes, the middle circle illustrates the GC content along consensus plastomes, and the inner circle illustrates nucleotide diversity (Pi) along the consensus plastome

A total of 111 genes were annotated in the *Paeonia* plastomes, including 78 protein-encoding genes, 29 tRNA-encoding genes and four rRNA-encoding genes (Table 3; Fig. 4). Based on their functions, all genes were classified into three groups: those related to plastome replication, those involved in photosynthesis, and those with other functions. Duplicate copies of genes were found in the *Paeonia* plastomes, with 20 genes having two copies in at least one accession, while 89 genes had only one copy. Most genes (107/111, including 74 protein-encoding genes, four rRNA-encoding genes and 29 tRNA-encoding genes) were consistent among all accessions, while four genes (*rpl22*, *rps3*, *rps19* and *ycf1*) showed different copy numbers among accessions (Supplementary Table 1).

**Table 3:** Genes annotated in *Paeonia* plastomes

Category	Gene functions	Names of genes
Self-replication	DNA-dependent RNA polymerase	*rpoA*, *B*, *C1*, *C2*
Self-replication	Large subunit of ribosomal proteins	*rpl2*, *14*, *16*, *20*, *22*, *23*, *33*, *36*
Self-replication	rRNA genes	*rrn16*, *rrn23*, *rrn4.5*, *rrn5*
Self-replication	Small subunit of ribosomal proteins	*rps11*, *rps12*, *rps14*, *rps15*, *rps16*, *rps18*, *rps19*, *rps2*, *rps3*, *rps4*, *rps7*, *rps8*
Self-replication	tRNA genes	*trnA-UGC*, *trnC-GCA*, *trnD-GUC*, *trnE-UUC*, *trnF-GAA*, *trnG-GCC*, *trnG-UCC*, *trnH-GUG*, *trnI-CAU*, *trnI-GAU*, *trnK-UUU*, *trnL-CAA*, *trnL-UAA*, *trnL-UAG*, *trnM-CAU*, *trnN-GUU*, *trnP-GGG*, *trnQ-UUG*, *trnR-ACG*, *trnR-UCU*, *trnS-GCU*, *trnS-GGA*, *trnS-UGA*, *trnT-GGU*, *trnT-UGU*, *trnV-GAC*, *trnV-UAC*, *trnW-CCA*, *trnY-GUA*
Photosynthesis	ATP synthase	*atpA*, *B*, *E*, *F*, *H*, *I*
Photosynthesis	Cytochrome b6/f complex	*petA*, *B*, *D*, *G*, *L*, *N*
Photosynthesis	NADH oxidoreductase	*ndhA*, *B*, *C*, *D*, *E*, *F*, *G*, *H*, *I*, *J*, *K*
Photosynthesis	photosystem assembly factor	*pafI*, *pafII*
Photosynthesis	Photosystem I	*psaA*, *B*, *C*, *I*, *J*
Photosynthesis	Photosystem II	*psbA*, *B*, *C*, *D*, *E*, *F*, *H*, *I*, *J*, *K*, *L*, *M*, *T*, *Z*
Photosynthesis	Rubisco	*rbcL*
Other genes	c-Type cytochrome synthesis gene	*ccsA*
Other genes	Conserved open reading frames	*ycf1*, *2*
Other genes	Envelope membrane protein	*cemA*
Other genes	Maturase	*matK*
Other genes	Protease	*clpP1*
Other genes	Subunit acetyl-CoA-carboxylase	*accD*

#### Codon usage bias

Codon usage bias was analyzed in all 78 protein-coding CDSs. The average RSCU values are listed in Supplementary Table 2. Thirty-one codons had a mean RSCU value greater than 1, 29 of which included A or U at the third position, indicating a preference for A and U bases in *Paeonia* codons. Based on the RSCU value, UUA, GCU and AGA were identified as the optimal codons in *Paeonia* plastomes. The mean ENC value of 78 CDS ranged from 25.29 (*rpl36*) to 61 (*rps18* and *rpl22*) (Supplementary Fig. 3), suggesting a high variance of codon usage bias. Five genes (*rpl36*, *petN*, *psbI*, *rpl33* and *psbJ*) had a mean ENC less than 35, indicating strong codon usage bias, while most genes (56/79) had a mean value greater than 45 and exhibited weak codon usage bias.

#### Selection analysis

Based on the results of the FUBAR and FEL models in HyPhy, most genes (52/78) (Supplementary Table 3) had sites under diversifying positive selection in at least one model. Out of 17 genes identified in both models, three were self-replication genes (out of 24), nine were photosynthesis genes (out of 46), and five were genes with other functions (out of 8). Episodic positive diversifying selection in two cultivar groups (MOUT_CULT and PAEO_CULT) was analyzed using the MEME model. In the MOUT_CULT group, no genes (*p* < 0.05) were identified while in the PAEO_CULT group, seven genes (one gene with another function (*ycf1*), four photosynthesis-related genes (*petA*, *psaA*, *psaB* and *rbcL*) and two self-replication genes (*rpoB*, *rps14*)) were found to have one-six significant loci (*p* < 0.05) under positive selection (Supplementary Table 4).

### Genetic diversity and phylogeny

#### Nucleotide diversity and promising DNA barcode regions

In the 63 analyzed plastomes, no genome rearrangement was detected by Mauve and the entire plastome was identified as a locally collinear block (Supplementary Fig. 4). Additionally, a sequence matrix of 152,188 bases was generated for the 63 accessions through alignment and trimmed by MAFFT and TrimAl, and a consensus plastome with a length of 152,188 bases was generated by seqinr. The nucleotide diversity (Pi value) of the five groups and all accessions’ datasets along the plastome was calculated based on the sequence matrix (Fig. 4), which demonstrates lower nucleotide diversity in the IR regions than the LSC/SSC regions. The Pi value calculated for the entire plastome indicates that natural accessions have higher nucleotide diversity (0.0020 for MOUT_WILD and 0.0018 for PAEO_WILD) than cultivars, which exhibited lower nucleotide diversity (0.0015 for MOUT_CULT and 0.0001 for PAEO_CULT). Nucleotide regions with high diversity were identified by comparing Pi values using a sliding window approach. The top three regions, including two intergenic regions (*ycf1*-*trnI_CAU* with a Pi value of 0.015 and *rrn23*-*trnR_CAU* with a Pi value of 0.011) and one genic region (within *ndhH*, 0.012) exhibited the highest diversity. Other regions that are promising for their development as DNA barcodes are listed in Supplementary Tables 5, notably the intergenic region between *ycf3* and *psbA* which demonstrated high nucleotide diversity in PAEO_CULT.

#### Genetic diversity and structure based on molecular markers

From the 63 plastomes, a total of 32 SSR markers and 4476 SNPs were identified. These were then used to investigate the accessions’ genetic structure. Analysis of both marker types revealed similar patterns of genetic diversity among the 63 accessions (Table 4). The expected number of multilocus genotypes (*eMLG*, a measure of genotype abundance) was much lower in PAEO_CULT than in the remaining three groups, which exhibited similar levels of *eMLG*. Similarly, the genotype diversity index (including *H*, *G* and *λ*) and evenness index (*E5*) showed similar patterns among the four groups (Table 4).

**Table 4:** Genotype diversity of two types molecular markers on five groups of the *Paeonia* plastome

Marker	Group	*N*	*eMLG*	*H*	*G*	*λ*	*E5*
SSR	MOUT_CULT	8	6	1.73	5.33	0.81	0.93
	MOUT_WILD	8	8	2.08	8.00	0.88	1
	PAEO_CULT	8	5	1.39	3.20	0.69	0.73
	PAEO_WILD	8	8	2.08	8.00	0.88	1
	Whole	32	26	3.13	18.29	0.95	0.79
SNP	MOUT_CULT	8	7	1.91	6.40	0.84	0.94
	MOUT_WILD	8	8	2.08	8.00	0.88	1
	PAEO_CULT	8	4	1.07	2.29	0.56	0.67
	PAEO_WILD	8	8	2.08	8.00	0.88	1
	Whole	32	9	3.09	16.00	0.94	0.72

*E5*, genotype evenness; *eMLG*, the expected number of multilocus genotypes at the lowest common sample size; *H*, Shannon-Weiner index; *G*, Stoddard and Taylor’s index; *λ*, Simpson’s index; *N*, sample size

**Fig. 5:**
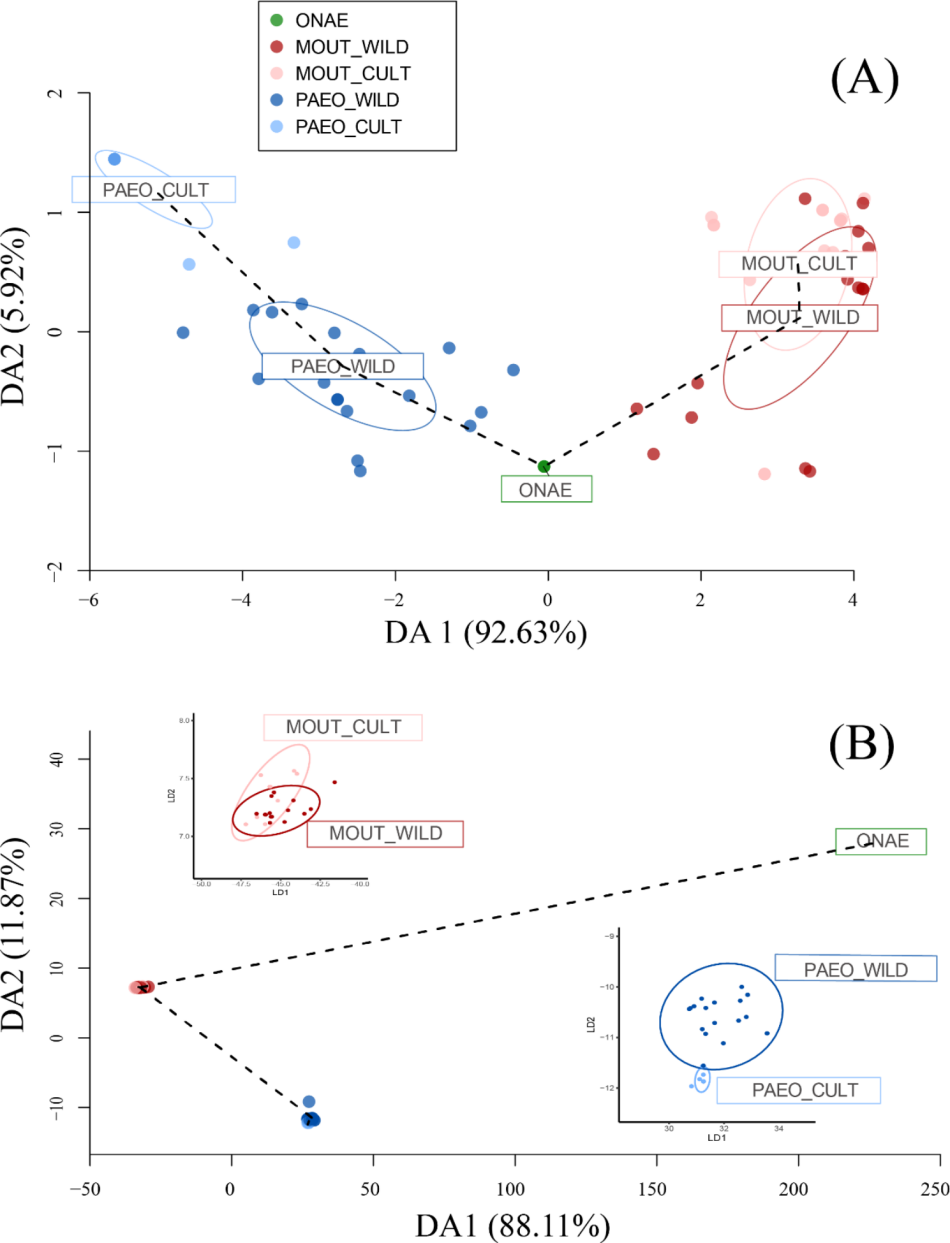
The distribution of five groups on the first 2 discriminant axes of DAPC. (**A**) results based on SSR; (**B**) results based on SNP. Both markers (SSR and SNP) could effectively distinguish the three sections, while SNP revealed much longer distances between sect. *Onaepia* with the two other sections than SSR

The DAPC results showed that both marker types effectively distinguished the three sections, although SNPs revealed longer distances between sect. *Onaepia* and the other two sections than SSRs (Fig. 5). The minimum spanning tree based on SSRs indicated that sect. *Onaepia* was located between the other two sections, while SNPs revealed that sect. *Onaepia* had a shorter distance to sect. *Moutan* than to sect. *Paeonia*. Both marker methods effectively distinguished PAEO_CULT from PAEO_WILD, but could not differentiate MOUT_CULT from MOUT_WILD. A phylogenetic analysis was used to assess the ability of both markers to delimit species. The results showed that SSRs only effectively delimited five species (*P. brownii*, *P. obovata*, *P. mairei*, *P. jishanensis* and *P. qiui*) whereas SNPs effectively delimited all species except for three (*P. veitchii*, *P. delavayi* and *P. rockii*) (Supplementary Fig. 5).

#### Phylogenetics of ***Paeonia*** based on plastomes

**Fig. 6:**
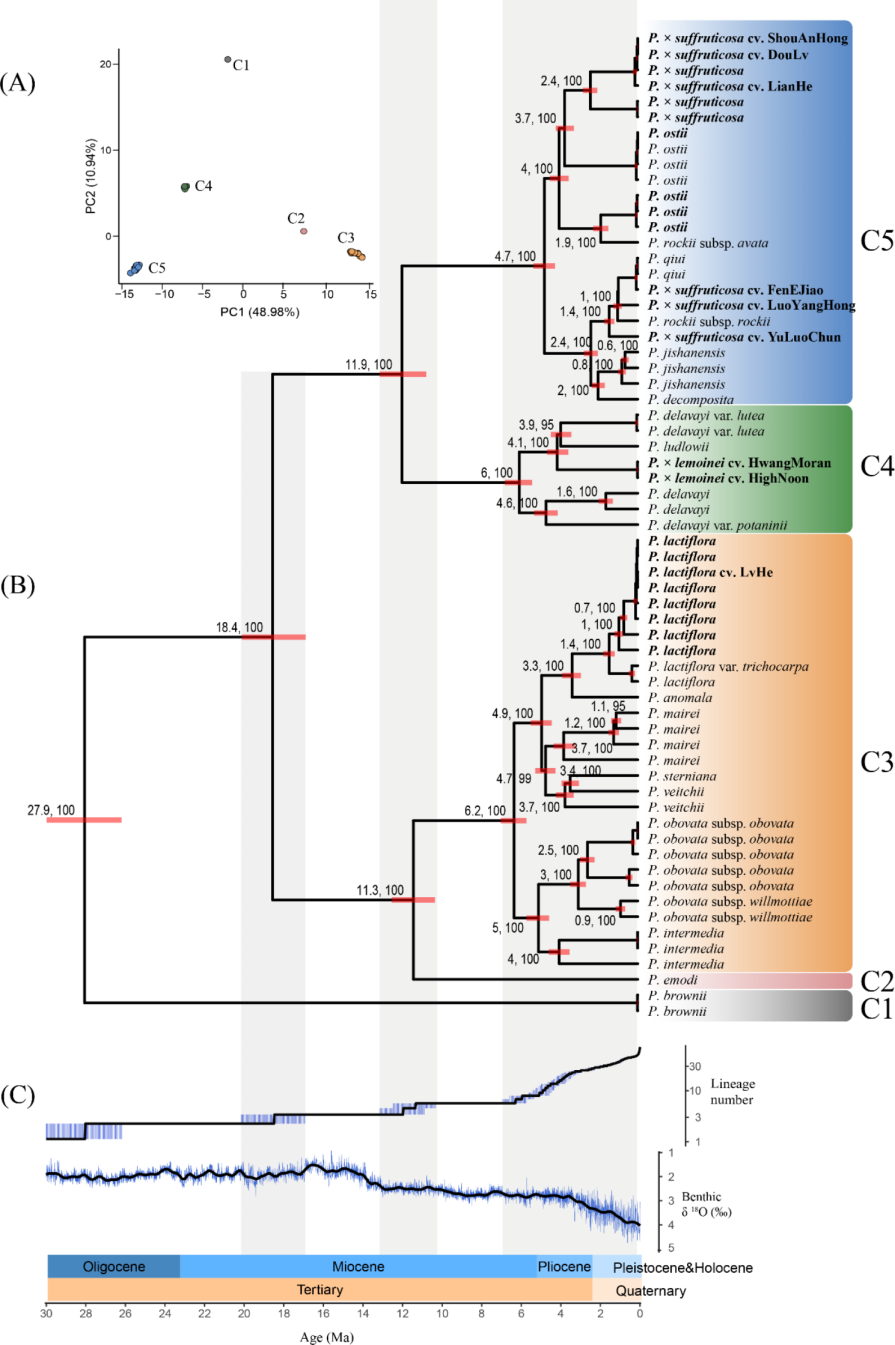
Results of Bayesian evolutionary analysis. (**A**) Distribution of 63 plastomes on the first 2 component based on 4476 SNPs; (**B**) maximum clade credibility tree with divergence time of 63 accessions; (**C**) lineage size and global temperature (inferred by the content of benthic δ ^18^O) changes along ages. Bayesian phylogeny revealed that *P. brownii* firstly diverged from other accessions, and that all accessions could be divided into five branches (C1-C5), corresponding to the five clusters of PCA results based on SNPs

PCA results based on SNPs (Fig. 6A) revealed five distinct clusters of all accessions, corresponding to the five branches revealed in the MCC tree (Fig. 6B). These clustering results provide support for the current taxonomic treatment of the three sections in *Paeonia*. The MCC tree showed that sect. *Onaepia* diverged from the two other Sects. 26–30 million years ago (Ma), followed by three periods of speciation. The first divergence occurred between sect. *Paeonia* and *Moutan* 17–20 Ma. The second period, which took place 11–13 Ma, resulted in the divergence of both sections into two branches. One of the branches is now distributed in pan-Himalaya while the other is widely distributed throughout East Asia. This period was followed by a sharp temperature drift, as shown in Fig. 6C. The last period, which spanned from 6.5 Ma to the present, resulted in the majority of the currently known species. Remarkably, this period coincided with a continuous decrease in global temperatures.

## Discussion

### Plastome evolution in ***Paeonia***

Since the sequencing of the first plastome in the 1980s [51], thousands of plastomes from land plant species have been reported over the past three decades, revealing abundant diversity in dynamic structure and content [11]. These plastomes have made a significant contribution to our understanding of both interspecies and intraspecies evolutionary history. With 10 newly reported *Paeonia* plastome assemblies, a total of 63 accessions were subjected to comprehensive analyses in this research to explore the plastome evolutionary pattern in *Paeonia*. Despite variations mainly attributed to IR variation and nucleotide diversity, the plastome structure was found to be conserved in *Paeonia*, with no rearrangements detected among all accessions, as is typically observed in most angiosperm genera [52],

IR contraction or expansion can result in gene loss or gain, and can also affect the dosage of genes located within them [13]. Genes located in IR undergo copy-dependent repair and experience doubling dosage affects, which can help stabilize and strengthen their biological function [52]. In this research, four accessions exhibited IR contraction, resulting in the loss of a complete copy of *rpl2*. Conversely, two accessions showed IR expansion, resulting in a duplicate copy of three genes (*rps19*, *rpl22* and *rps3*). These genes are crucial for ribosome assembly and protein synthesis in the plastid, so any variation in dosage may impact leaf development and overall plant growth [53]. Notably, the two accessions showing IR expansion were samples from the margin of distribution of their respective species. *P. delavayi* (KY817591) was collected from Tibet, which has a higher altitude than other *P. delavayi* accessions, which were collected from Yunnan province in China. Whereas *P. lactiflora* (MN868412) was collected from southern China, most other *P. lactiflora* accessions were collected from northern China. IR expansion followed by gene duplication may have contributed to the ability of these two accessions to withstand environmental pressures, such as high altitude or temperature [54].

Despite the typically stable nucleotide content and highly conserved gene structure of chloroplast genomes, mutation hotspots can still occur [55]. In this research, 10 potential loci were identified that could serve as DNA barcodes for future research in *Paeonia*. Among them, the *ycf1* region exhibited the highest level of nucleotide diversity. The high variability of the *ycf1* region has also been observed in other genera [56, 57], making it a recommended plastid barcode for land plants [58]. In our study, the Pi value showing the longest genetic distance (~ 0.01) among the 63 accessions was between *P. brownii* and *P. obovata*. Notably, this distance is considerably lower than in other genera like *Musa* (0.03) [59] and *Miscanthus* (0.05) [60]). As the only genus in the Paeoniaceae and a single lineage within the Saxifragales, *Paeonia* is often considered to be ancient due to its possession of ancient biological traits such as the centrifugal development of stamens [1]. However, this research suggests that while it has an ancient origin, it diverged relatively late. This is supported by the low genetic diversity observed in our study, which suggests that ancient *Paeonia* branches may have experienced wide extinction events, while currently existing species may have arisen from recent speciation events. High hybridization affinity among *Moutan* species and reports of intersectional crosses [3] suggest that reproductive isolation among *Paeonia* species may be relatively weak. Collectively, those observations suggest close genetic relationships among *Paeonia* species, or at least among those found in East Asia.

### Plastome reveals the domestication history of ***Paeonia*** cultivars

Genetic analysis is essential for cultivar breeding and utilization, and plastomes can significantly contribute to these efforts, particularly in the fields of pedigree analysis [17, 61], evaluation of genetic diversity [53], and exploration of domestication [18]. To compare the genetic structure of cultivated and natural *Paeonia* accessions, we manually clustered 63 accessions into five groups. However, these groups did not align with the five clusters generated by Bayesian phylogenetics and PCA. This is not surprising given the ancient nature of the *Paeonia* genus [1]. Compared to the five deep clusters revealed by phylogenetics and PCA, genetic variation between cultivated and natural accessions was relatively minor. Cultivated and natural accessions were grouped despite these inconsistencies because this approach might provide valuable insight into the domestication of cultivars.

The origin of the first reported tree peony species, *P. suffruticosa*, is a topic of debate [3]. According to some researchers, it is a hybrid formed by repeated hybridization among several species in sect. *Moutan*, based on both morphological and DNA markers (ADLP and RAPD) [62, 63]. Others, however, have argued that *P. suffruticosa* is not a hybrid but rather a cultivated variant of *P. cathayana* [6, 64]. Our results indicate that nine *P. suffruticosa* accessions were divided into two groups with different maternal origins, suggesting that *P. suffruticosa* is a hybrid complex resulting from multiple hybridizations. Additionally, our results revealed that *P. ostii* exhibits high genetic diversity, branching into three clades that include six *P. suffruticosa* cultivars whose maternal origin was traced back to *P. cathayana* [6]. This finding suggests that *P. cathayana* may be a specialized form of *P. ostii*, supported by the fact that they share a similar nuclear genome [6]. A previous study suggested that the cultivar ‘Luo Yang Hong’ was maternally inherited from species such as *P. rockii* and *P. qiui*, but the precise maternal origin was unclear [6]. Our results indicate that ‘Luo Yang Hong’ was maternally inherited from *P. rockii* subsp. *rockii*, rather than *P. qiui*, while cultivar ‘Fen E Jiao’ may have been inherited from *P. qiui*. These findings underscore the utility of the entire plastome in revealing high-resolution domestication history in *Paeonia*.

Chinese herbaceous peony cultivars were reported to have originated from wild *P. lactiflora*, without hybridizing with other species [65]. Our results are consistent with that finding. Additionally, all herbaceous cultivars were clustered into a monophonic group that was independent of wild *P. lactiflora* accessions. These results suggest that herbaceous peony cultivars were likely introduced from the wild on a single occasion and subsequently underwent a common domestication process. This may have resulted in the low genetic diversity of PAEO_CULT, highlighting the importance of introducing other wild *Paeonia* germplasm. Seven genes were identified as being under positive selection in herbaceous peony cultivars, including four photosynthesis-related genes: *petA*, *psaA*, *psaB* and *rbcL*. The *petA* gene encodes cytochrome f, a protein that plays a critical role in electron transfer during photosynthesis [66]. The *psaA* and *psaB* genes encode the large core subunit of photosystem I, which is involved in a variety of metabolic and physiological responses in plants [67]. The *rbcL* gene encodes the large subunit of Rubisco, a key enzyme in CO_2_ assimilation [68]. The positive selection of these genes, all of which are crucial for photosynthesis, may have contributed to the strong photosynthetic ability of Chinese herbaceous peony cultivars, potentially explaining their wide ecological range throughout China [1, 3]. However, further research is needed to fully understand the relationship between positive selection of photosynthesis-related genes and the ecological success of Chinese herbaceous peony cultivars.

### Plastome reveals the evolutionary history of ***Paeonia***

*Paeonia* has undergone frequent instances of polyploidization and hybridization [7]. These have made it challenging to fully reconstruct the evolutionary history of this genus. However, technological advancements have made progress possible. In this study, several notable discoveries were made with the aid of the plastome.

The first issue in phylogenetic and taxonomic research of *Paeonia* may be how to deal with species in sect. *Onaepia*. In contrast to previous studies [7, 16], our findings suggest that sect. *Onaepia* represents the first branch to diverge from the ancient *Paeonia* lineage. This divergence likely occurred around 26–30 Ma, during a period of increased dispersal from Asia to North America associated with the late Oligocene warming [69]. Other closely related lineages, including *Deutzia* [70], *Saxifraga* [71] and *Darmera* [72], also experienced divergence around that time, suggesting that sect. *Onaepia* arose independently from Asian branches, and that at least two separate herbacelizing events occurred in *Paeonia*.

Our research revealed that *P. delavayi* var. *lutea* has a closer maternal relationship with *P. ludlowii* than with two other variants (*P. delavayi* var. *delavayi* and var. *potaninii*), indicating that the *P. delavayi* plastome is paraphyletic. However, prior assessment of the nuclear genome indicated that *P. delavayi* is monophyletic [7, 9]. These conflicting phylogenetic signals may be explained by plastid capture [73]. In this process, ancient *P. delavayi* captured the plastome from *P. ludlowii* and generated *P. delavayi* var. *lutea*. Another similar bi-species complex exists with *P. veitchii* and *P. sterniana*. Our results indicate that *P. veitchii* also has a paraphyletic plastome, consistent with findings from its nuclear genome [7]. This suggests that *P. sterniana* may be a specialized form of *P. veitchii* and that the taxonomy of *P. veitchii* may need to be revised accordingly.

Another interesting issue is the consistency between the abrupt global cooling since the Pliocene and the rapid divergence of the *Paeonia* lineage (5.3 Ma, Fig. 5C). Abrupt global cooling during the Pliocene may have rendered the habitats of ancient peonies unsuitable [74], reducing their ability to survive and reproduce. However, the heterogenic landscapes of the Pan-Himalayan region may have provided suitable refuge, allowing for the survival of some ancient *Paeonia* lineages [75]. As a result, the surviving lineages might have diverged from each other and eventually resulted in the present *Paeonia* species. Thus, both paleoclimatic and geographic events may have contributed to the process of *Paeonia* diversification. However, further research is needed to fully understand the origin and dispersal routes of specific species.

## Conclusion

Utilizing 10 newly reported assemblies of *Paeonia* plastomes, a dataset covering all species in East Asia was generated. Based on this dataset, a comprehensive phylogenetic and comparative genomic analysis was performed. The results showed an overall conserved structure and low nucleotide variation among all plastomes, although several accessions exhibited IR expansion and contraction. These findings suggest that woody cultivars had multiple maternal origins although no plastome gene showed traces of selection via domestication. Conversely, herbaceous cultivars were only inherited from *P. lactiflora*. Several genes related to photosynthesis showed evidence of selection during domestication. The phylogenetic results validated the ability of plastomes to delimit species, revealed a consistency between *Paeonia* speciation and global paleoclimatic change, and supported an independent taxonomic treatment of sect. *Onaepia*. Collectively, these results provide a comprehensive set of valuable information for understanding the evolutionary and domestication history of *Paeonia* and are useful for the conservation and utilization of both natural and cultivated germplasm.

## Electronic supplementary material

Below is the link to the electronic supplementary material.

Supplementary Material 1

## Data Availability

The 10 newly reported plastomes are openly available in NCBI at the GenBank database with accession numbers OP324584-OP324593. Additional data are provided as supporting information in this article.
