# Supplementary material for: The plastome reveals new insights into the evolutionary and domestication history of peonies in East Asia
Source: BMC Plant Biol. 2023 May 8;23:243. doi: 10.1186/s12870-023-04246-3 (PMC10165817; doi:10.1186/s12870-023-04246-3)
Supplement: Supplementary file 1 — Supplementary Material 1 [file 12870_2023_4246_MOESM1_ESM.docx]

Supplementary Figure legends


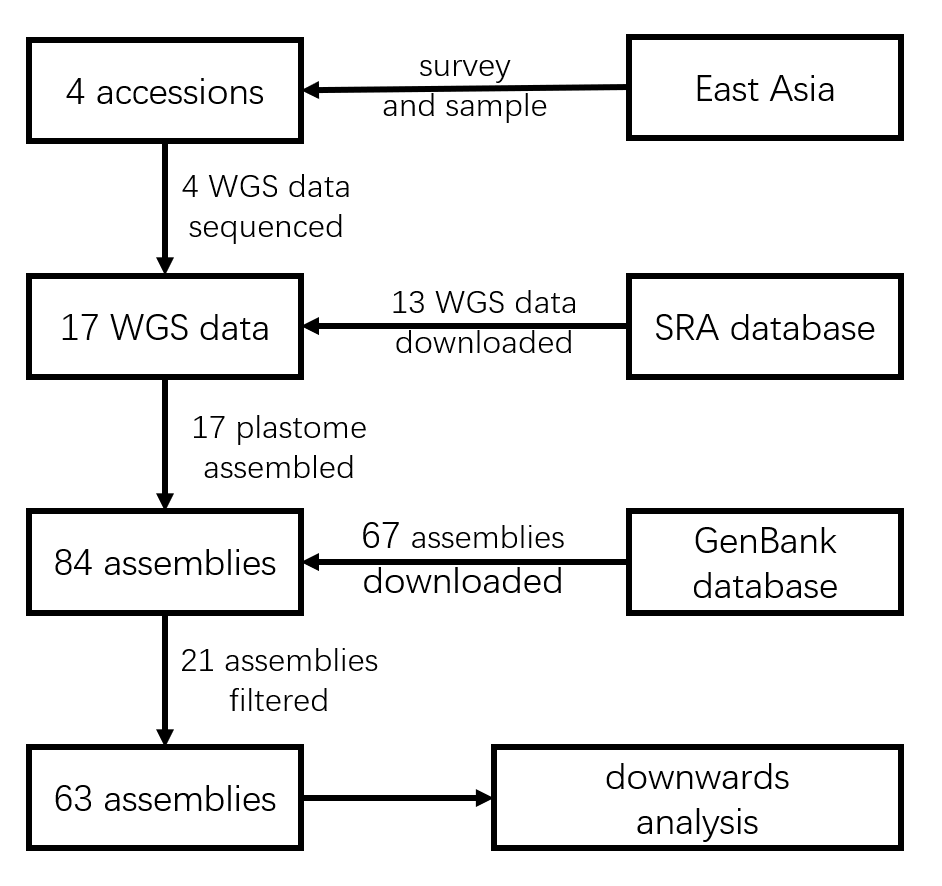


Supplementary Figure 1. The data preparation process of 63 accessions.


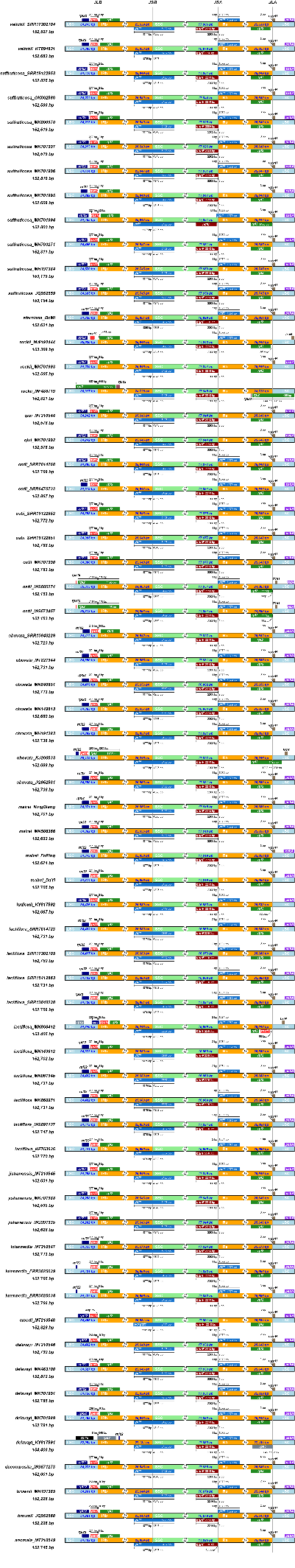


Supplementary Figure 2. Full version of inverted repeat region (IR) distribution and structure of 63 *Paeonia* plastomes. A conserved structure was observed among all plastomes, although several demonstrated IR expansion and contraction.


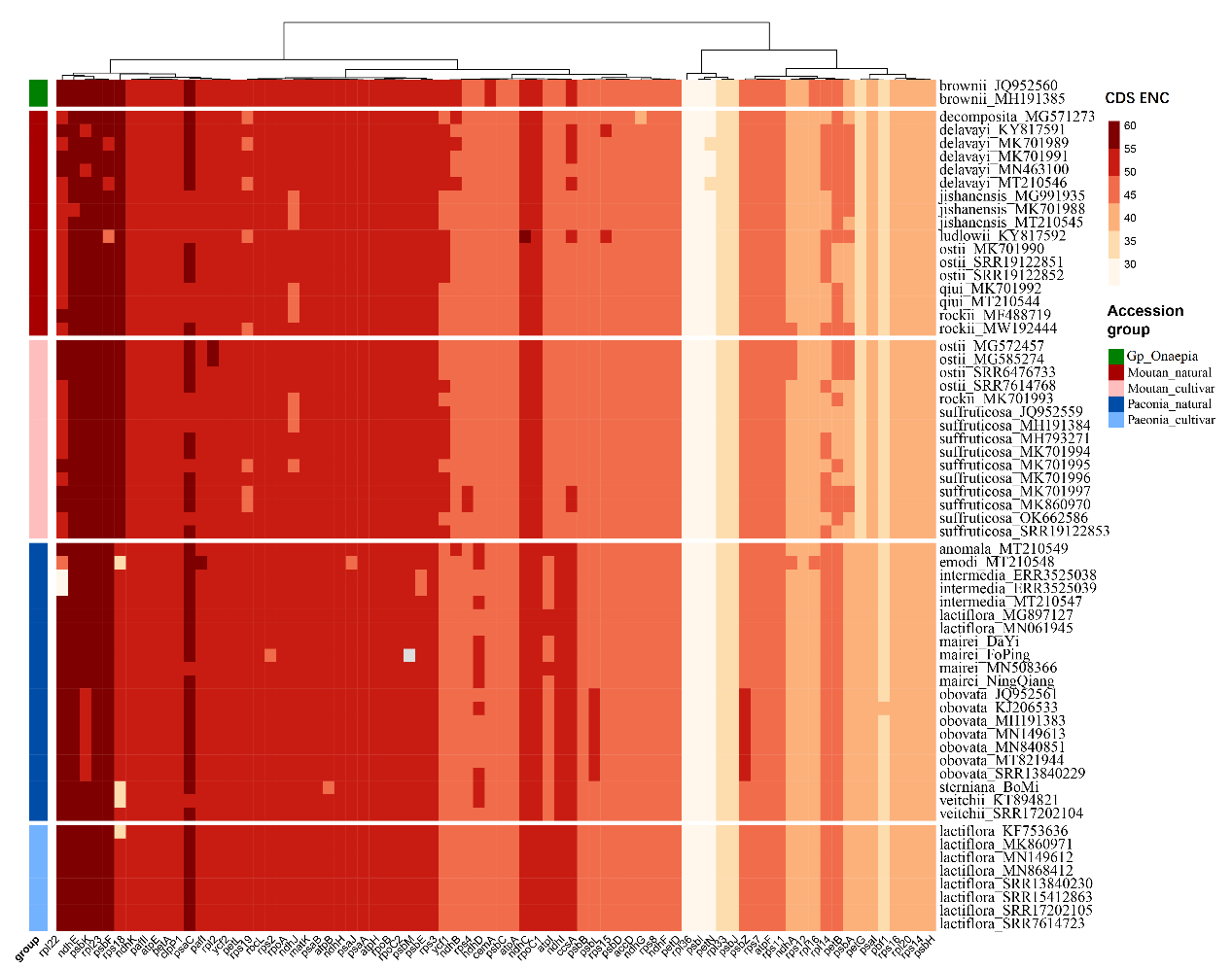


Supplementary Figure 3. Effective number of codons of all protein-coding CDSs in all plastomes. Most CDSs (56) had ENC > 45, while five CDSs had ENC < 35.


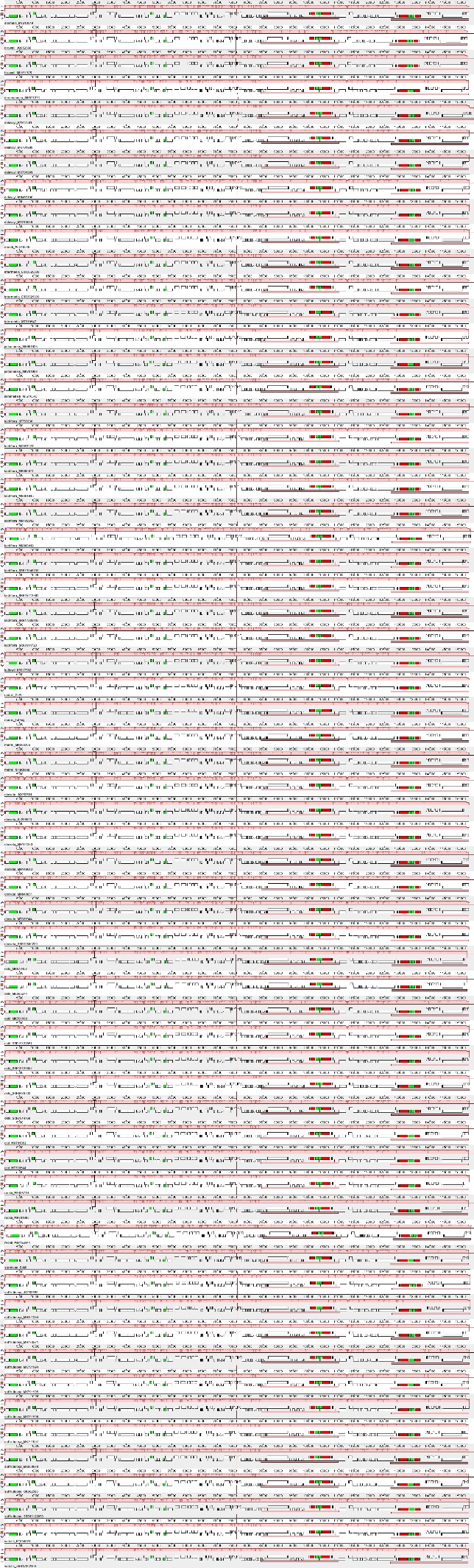


Supplementary Figure 4. Multiple sequence alignment of 63 *Paeonia* plastomes using Mauve. No genetic rearrangements were detected, and the whole plastome of each accession was identified as a synonymous block.


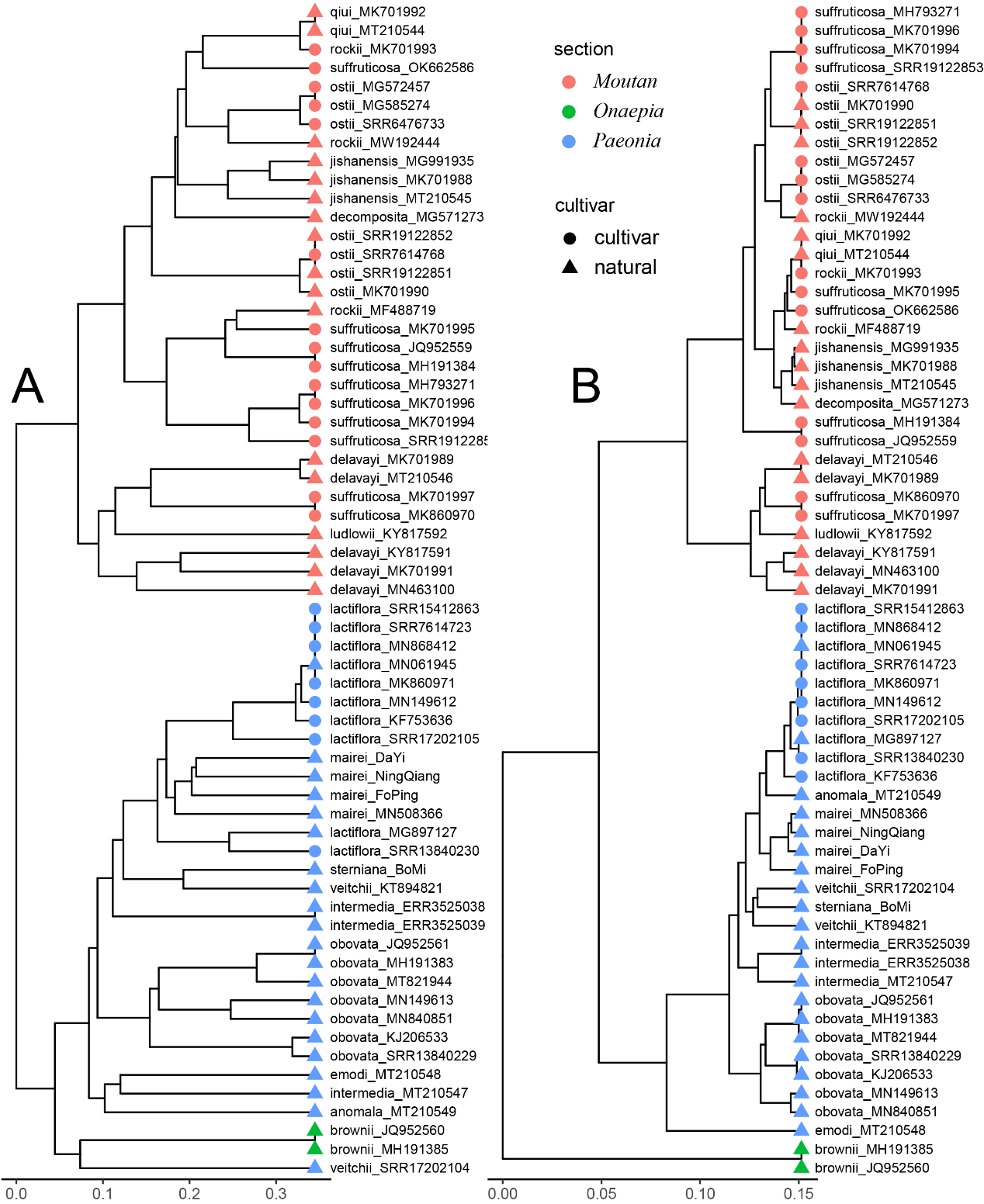


Supplementary Figure 5. Phylogeny based on SSR (A) and SNP (B) markers. Both markers discriminated section *Paeonia* from section *Moutan*, but SSR had a low ability to delimit species while SNP effectively delimited all species (except for *P. veitchii*, *P. delavayi* and *P. rockii*).

Supplementary Tables

| Supplementary Table 5. Top 10 hotspots in the *Paeonia* plastome that are promising for the development of a barcode | | | | | | |  |
| --- | --- | --- | --- | --- | --- | --- | --- |
| idx | start | end | Nei value among whole accessions | type | gene nearest by start position | gene nearest by end position | gene inside the region |
| 1 | 124001 | 125000 | 0.011938251 | genic region | ycf1 | ycf1 |  |
| 2 | 109501 | 110500 | 0.011648929 | genic region | ndhF | ndhF |  |
| 3 | 123501 | 124500 | 0.011436021 | genic region | ycf1 | ycf1 |  |
| 4 | 59001 | 60000 | 0.010585102 | genic region | psaI | psbJ |  |
| 5 | 63001 | 64000 | 0.010442353 | intergenic region | petA | accD |  |
| 6 | 110001 | 111000 | 0.010357923 | genic region | ndhF | ndhF |  |
| 7 | 111501 | 112500 | 0.010343241 | intergenic region | ndhF | ccsA |  |
| 8 | 112001 | 113000 | 0.010173676 | intergenic region | ndhF | ccsA |  |
| 9 | 6501 | 7500 | 0.010066022 | intergenic region | rps16 | psbK |  |
| 10 | 65501 | 66500 | 0.009997956 | combined_region | petL | psaJ | petG |
